# Cell type-specific autophagy in human leukocytes: signatures of aging, sex, and nutrient restriction

**DOI:** 10.1080/27694127.2025.2543560

**Published:** 2025-08-17

**Authors:** Linh VP Dang, Timothy J Sargeant

**Affiliations:** a Lysosomal Health in Ageing, Lifelong Health, South Australian Health and Medical Research Institute, Adelaide, SA, Australia; b Adelaide Medical School, University of Adelaide, Adelaide, SA, Australia

**Keywords:** Aging, autophagy, autophagic flux, LC3B-II, nutrition, PBMCs, sex

## Abstract

Macroautophagy (referred to here as autophagy) is thought to play a critical role in aging and age-related disease, making it a priority for development of targeted human therapies. We developed a flow cytometry-based method to measure autophagic flux in 19 subpopulations from whole blood, using chloroquine (CQ) to inhibit lysosomal degradation, and the autophagy protein MAP1LC3B (microtubule associated protein 1 light chain 3 beta) isoform II/LC3B-II to measure autophagic flux (the acquisition and degradation of autophagic cargo over time). Autophagic flux varies by cell type and is higher in whole blood compared with RPMI culture media. Basal autophagic flux shows sex- and age-specific variations. Further, monocytes, but not T cells, respond robustly to amino acid starvation by increasing autophagy, with older individuals exhibiting stronger responses, particularly in non-classical monocytes. These findings underscore the importance of cell type-specific autophagy measurements to understand the effects of aging, sex and nutrition, to develop targeted interventions for age-related diseases.

Autophagy is a nutrient- and stress-response essential for cellular homeostasis by providing nutrients during starvation and clearing damage or pathogens. Preclinically, impaired autophagy is associated with aging and age-related diseases, and is modifiable by drugs such as mTOR inhibitors or nutrient restriction. Accurately measuring physiological autophagy and especially *in vivo* is critical for guiding human disease interventions. We previously developed a method to assess the autophagic flux in PBMCs (peripheral blood mononuclear cells) using western blot and enzyme-linked immunosorbent assay (ELISA), by analyzing the difference in LC3B-II abundance between CQ-treated and untreated samples. PBMCs, however, consist of different cell subpopulations (e.g., *T*-, B-, and NK-cells, and monocytes), each with distinct roles in immune surveillance and tissue homeostasis. Cell-specific autophagy could be crucial in aging, disease, and interventions such as exercise or nutrition, and represents a way to assess target engagement for human autophagy-based interventions. To address this issue we developed a flow cytometry-based method to measure autophagic flux in 19 cell subpopulations, which we reported in our recent manuscript^[1]^. Whole blood was incubated with or without CQ for 1 h, stained with extracellular markers to identify cell types, and washed with 0.05% saponin to remove LC3B-I before staining for LC3B-II using fluorochrome-conjugated antibody. Flux was calculated as the difference in Geometric Mean Fluorescence Intensity (MFI) of LC3B-II between CQ-treated and -untreated samples:

ΔMFI (LC3B-II) = (MFI LC3B-II with CQ) – (MFI LC3B-II without CQ)

Our data indicate that cell-specific autophagic flux is higher in whole blood compared to RPMI culture, underscoring the importance of using whole blood to measure autophagy in PBMCs and their populations, hinting at the importance of the physiological environment for autophagy. While the autophagic flux in each population varied, significant differences were observed within specific subpopulations: flux in non-classical monocytes was higher than in classical and intermediate monocytes; NK-, B-, and T-cells all displayed subpopulation-specific autophagy levels as well. These findings also suggest that shifts in subpopulation relative frequency can affect perception of flux at the PBMC level, emphasizing the importance of assessing cell type-specific autophagy.

We found autophagy also correlated with participant characteristics. Importantly, monocytes, particularly non-classical monocytes, from females exhibited significantly higher flux than in males. Given their role in vascular homeostasis, this finding may have relevance for sex differences in cardiovascular disease. Autophagic flux also positively correlated with age across most PBMC subpopulations, particularly in total PBMCs, monocytes, classical and intermediate, T cell subsets, and all NK cell subpopulations. This increase did not correlate with inflammatory cytokine levels, i.e. interleukin-1 and interleukin-6, suggesting the possibility of basal autophagy upregulation to counteract age-related cellular damage in young adults. However, older people and those with chronic disease may experience elevated inflammation, which could influence autophagy levels. Regardless, the reason for this increase remains speculative. A further challenge is identifying the most suitable cell type that reflects autophagic flux changes during aging. Long-lived T cells, especially memory T cells may be good indicators of aging, while relatively short-lived monocytes could be particularly informative in age-related diseases such as cardiovascular diseases.

Cell type-specific responses to nutrient starvation highlight the need to select appropriate cell types for nutritional studies: monocytes strongly induce autophagy during amino acid starvation in vitro, but their short lifespan may be best suited to short-term studies; autophagic flux in B- and NK cells also appeared to increase under starvation, though the difference was not significant. Larger studies are required. While NK cells, with lifespans of ~2 weeks, may be unsuitable for extended studies, B cells, with longer lifespans, could represent autophagy more consistently during prolonged interventions if robust nutrient responses are revealed with regards to autophagy.

Overall, our data highlight the importance of measuring cell type-specific autophagy in the context of aging, sex, and nutritional interventions, and potentially in age-related conditions. However, this study has limitations: CQ might induce conjugation of ATG8s to single membranes (CASM), potentially confusing results. Despite this, CQ remains the most suitable option for whole blood assays due to its water solubility and rapid cellular penetration, unlike other lysosomal inhibitors that have poor membrane permeability or require dimethyl sulfoxide, which causes hemolysis and may interfere with autophagy. Another limitation is uncertainty as to whether autophagy levels in blood accurately reflect those in other body tissues. Data from mice and *C. elegans* have shown inconsistent changes in autophagy with age. However, those models are not fully representative of human biology. Thus, it remains unclear which human tissues show similar age-related changes and whether blood-based measurements reliably reflect autophagy in other tissues.

Our paper shows the importance of analyzing physiological autophagy at the cell population level in humans. However, we need to clarify what is driving age-related increases in autophagy observed in leukocytes. We also need to understand and eliminate interference by CASM: this may be the identification of autophagy cargos that work robustly in human leukocytes and are not ATG8 proteins, the development of water-soluble lysosome inhibitors that do not cause CASM, or specific CASM inhibitors. We also need to understand whether leukocyte autophagy reflects autophagy in other tissues. These issues are challenging but addressing them will yield significant new insights and applications.

## Data Availability

Data sharing is not applicable to this article as no data were created or analyzed in this study.

## Abbreviations

CASM: conjugation of ATG8s to single membranes;
CQ: chloroquine;
ELISA: enzyme-linked immunosorbent assay;
MAP1LC3B: microtubule associated protein 1 light chain 3 beta;
NK: natural killer;
PBMCs: peripheral blood mononuclear cells.
